# Strong Antimicrobial and Healing Effects of Beta-Acids from Hops in Methicillin-Resistant *Staphylococcus aureus*-Infected External Wounds In Vivo

**DOI:** 10.3390/antibiotics10060708

**Published:** 2021-06-12

**Authors:** Radek Sleha, Vera Radochova, Jiri Malis, Alexander Mikyska, Milan Houska, Karel Krofta, Katerina Bogdanova, Sylva Janovska, Jaroslav Pejchal, Milan Kolar, Pavel Cermak, Pavel Bostik

**Affiliations:** 1 Department of Epidemiology, Faculty of Military Health Sciences, University of Defence, 500 01 Hradec Kralove, Czech Republic; radek.sleha@unob.cz (R.S.); vera.radochova@unob.cz (V.R.); jiri.malis@ftn.cz (J.M.); sylva.janovska@unob.cz (S.J.); 2 Thomayer Hospital, 110 00 Prague, Czech Republic; pavel.cermak@lf3.cuni.cz; 3 Research Institute of Brewing and Malting, 110 00 Prague, Czech Republic; mikyska@beerresearch.cz; 4 Food Research Institute, 110 00 Prague, Czech Republic; milan.houska@vupp.cz; 5 Hop Research Institute, 438 01 Zatec, Czech Republic; krofta@chizatec.cz; 6 Department of Microbiology, Faculty of Medicine and Dentistry, University Hospital, Palacky University, 771 47 Olomouc, Czech Republic; katerina.bogdanova@fnol.cz (K.B.); kolar@fnol.cz (M.K.); 7 Department of Toxicology and Military Pharmacy, Faculty of Military Health Sciences, University of Defence, 500 01 Hradec Kralove, Czech Republic; jaroslav.pejchal@unob.cz; 8 Institute of Clinical Microbiology, Faculty of Medicine in Hradec Kralove, Charles University and University Hospital, 500 05 Hradec Kralove, Czech Republic

**Keywords:** hops, methicillin-resistant, *Staphylococcus aureus*, infection, porcine model

## Abstract

*Staphylococcus* (*S.*) *aureus* is an important causative agent of wound infections with increasing incidence in the past decades. Specifically, the emergence of methicillin-resistant *S. aureus* (MRSA) causes serious problems, especially in nosocomial infections. Therefore, there is an urgent need to develop of alternative or supportive antimicrobial therapeutic modalities to meet these challenges. Purified compounds from hops have previously shown promising antimicrobial effects against MRSA isolates in vitro. In this study, purified beta-acids from hops were tested for their potential antimicrobial and healing properties using a porcine model of wounds infected by MRSA. The results show highly significant antimicrobial effects of the active substance in both the powder and Ambiderman-based application forms compared to both no-treatment control and treatment with Framycoin. Moreover, the macroscopic evaluation of the wounds during the treatment using the standardized Wound Healing Continuum indicated positive effects of the beta-acids on the overall wound healing. This is further supported by the microscopic data, which showed a clear improvement of the inflammatory parameters in the wounds treated by beta-acids. Thus, using the porcine model, we demonstrate significant therapeutic effects of hops compounds in the management of wounds infected by MRSA. Beta-acids from hops, therefore, represent a suitable candidate for the treatment of non-responsive nosocomial tissue infections by MRSA.

## 1. Introduction

*Staphylococcus (S.) aureus* is an etiologic agent of various infections in both humans and animals. These bacteria colonize the entry of the nasal cavity in up to 80% of the human population and can be found on other mucosal surfaces [1]. Its pathogenic potential is associated with a variety of diseases, ranging from minor skin infections to the life-threatening toxic shock syndrome. The skin and soft tissue infections (SSTI) caused by *S. aureus* represent one of the major current healthcare problems. SSTI are very often associated with inpatient care and *S. aureus* has become a major nosocomial pathogen worldwide [2,3].

For many years, the management of SSTI was accomplished using beta-lactam antimicrobial drugs that are effective against β-hemolytic streptococci. The emergence of antibiotic-resistant strains of *S. aureus,* commonly known as methicillin-resistant *S. aureus* (MRSA), has complicated the use of these standard treatment regimens. The increased prevalence of MRSA especially in nosocomial settings significantly increases the morbidity, mortality, length of stay, and cost burden. In addition, the utilization of standard antibiotic treatment schemes further induces bacterial resistance and can create an even higher burden for the patient [4]. To reverse this continually increasing bacterial resistance, effective non-classical antimicrobial treatment alternatives are urgently needed and have been investigated for several years. Among those, various hops extracts and individual hops compounds have shown an antimicrobial potential, and therefore, represent a useful solution for the treatment of SSTI. The biological activities of such compounds have been known for some time, including their antimicrobial and anti-inflammatory effects. The data from various laboratories, including ours, have shown potent in vitro antimicrobial activities of hops compounds (isolated from *Humulus lupulus* L.), such as xanthohumol or alfa- and beta-acids against many bacterial pathogens, such as MRSA, *Pseudomonas aeruginosa*, *Helicobacter pylori*, or *Clostridium* spp. [5,6,7]. With their minimal inhibitory concentrations being close to commonly used antibiotics, these compounds may represent useful treatment alternatives provided that they show these antimicrobial effects in vivo as well.

While various animal models may prove suitable for the in vivo studies of SSTI, the porcine model is commonly used in experimental wound healing and treatment experiments. The pig, therefore, is the animal of choice when developing a wound infection model due to its similarity of skin anatomy, physiology, and immune system to humans. Porcine models are well accepted as being one of the best for human dermal repair due to the similarities between porcine and human skin [8,9,10,11,12].

The purpose of this study was to analyze antimicrobial and therapeutic effects of natural compounds isolated from hops in the porcine model of MRSA-infected wounds.

## 2. Results

**Figure 1:**
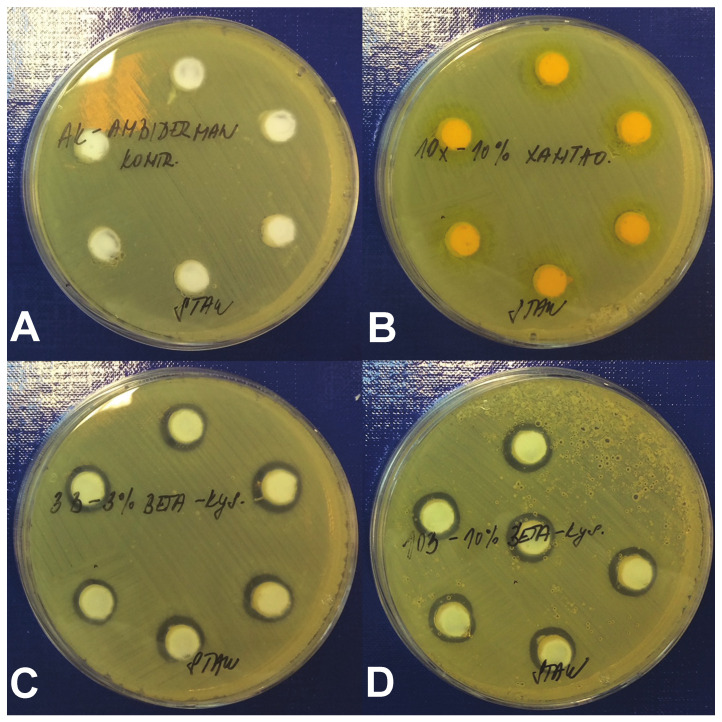
Antimicrobial activity of hop compounds in vitro. Agar diffusion test was performed using MRSA and compounds applied in Ambiderman base. (**A**) Ambiderman base only (negative control), (**B**) 10% xanthohumol, (**C**) 3% beta-acids and (**D**) 10% beta-acids. Inhibition zones.

Our previous data showed that both xanthohumol and beta-acids from hops possess strong antimicrobial activity against MRSA in vitro under the standard testing conditions [5,13]. However, when using antimicrobial substances in vivo, especially in wounds, binding of the tested compounds to the proteins secreted during the healing process may substantially modify their efficacy. An assessment using a modified agar diffusion method in the presence of BSA was, therefore, performed to quantitate the antimicrobial effect of the hop compounds in the presence of increased concentrations of proteins (Figure 1).

The diameters of inhibition zones for individual hops compounds and a hydrophilic base—Ambiderman control—are presented in Table 1. Indeed, the presence of BSA substantially decreased the antimicrobial activities of the tested compounds. The growth inhibition of MRSA was no longer detectable in xanthohumol at any concentration used. Only beta-acids retained the antimicrobial effect, but it was achieved at higher concentrations than in standard in vitro testing conditions without the addition of BSA [13]. The tested concentrations—3%, 5%, and 10%—did not show any significant differences in the diameters of the inhibition zones. Based on these results, the further evaluation of xanthohumol in the in vivo porcine model was not performed.

The experimental porcine model was subsequently established to evaluate the antibacterial and healing effects of purified hops compounds in vivo. External wounds were created and experimentally infected with a quantified dose of bacterial suspension of MRSA. The beta-acids from hops were applied to the wounds on days 3 and 7 after infection either in a powder form or in Ambiderman base during standard surgical dressing. Framycoin was used as a positive control due to its efficacy against a wide spectrum of bacteria and general suitability in the porcine model [14]. Bacterial load in the wounds, their size and macroscopic appearance on the standardized Wound Healing Continuum [15] were assessed.

**Figure 2:**
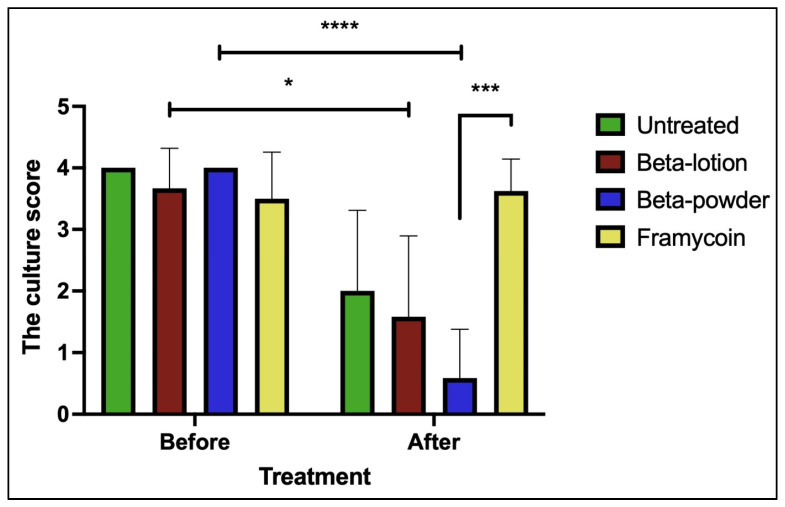
The effects of beta-acids on MRSA growth. Quantitation of MRSA in wounds before and on day 10 (after) of the treatment protocol is shown. Statistical significance (nonparametric Kruskal–Wallis test, multiple comparison test): * *p* < 0.05, *** *p* < 0.001, **** *p* < 0.0001.

The first quantitation of MRSA in the wounds was performed on day 3 post-inoculation before the application of the first treatment to verify the infection of the wounds and then on days 7 and 10 to record the anti-microbial effect of the applied substances. The effects of beta-acids on the growth of MRSA are shown in Figure 2.

All the wounds infected with MRSA showed a comparable bacterial growth before the treatments started. While some decrease in the culture score could be observed even in the untreated wounds, highly significant reductions of the bacterial growth were detected in the wounds treated with beta-acids in both the lotion and powder forms when compared to the pre-treatment levels (*p* < 0.05 and *p* < 0.0001, respectively). In addition, the beta-acid powder treatment exhibited a highly significant antimicrobial effect when compared to Framycoin (*p* < 0.001), which had no effect on the MRSA growth. Comparison of the bacterial growth levels in beta-acid treated wounds to the untreated ones revealed a clear positive effect of the beta-acid powder, although it was not statistically significant. These data show that the beta-acids exhibit a strong antimicrobial effect against the MRSA and this effect is pronounced the most when the substances are applied in the powder form.

**Figure 3:**
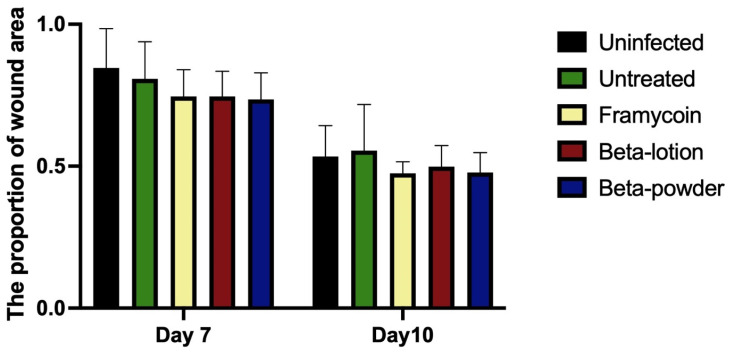
The effect of antibacterial treatments on the wound area. Areas of the individual wounds on days 7 and 10 were related to the respective wound area at the beginning of the experiment. The data are presented as means and standard deviations using the nonparametric Kruskal–Wallis test and multiple comparison test.

To assess the effect of the compounds on wound healing, the areas of individual wounds were compared first as the general indicator of the healing process. Figure 3 shows that wound areas decreased during the experiment regardless of the infection or treatment used. Such results point to the innately high healing potential of porcine skin.

**Figure 4:**
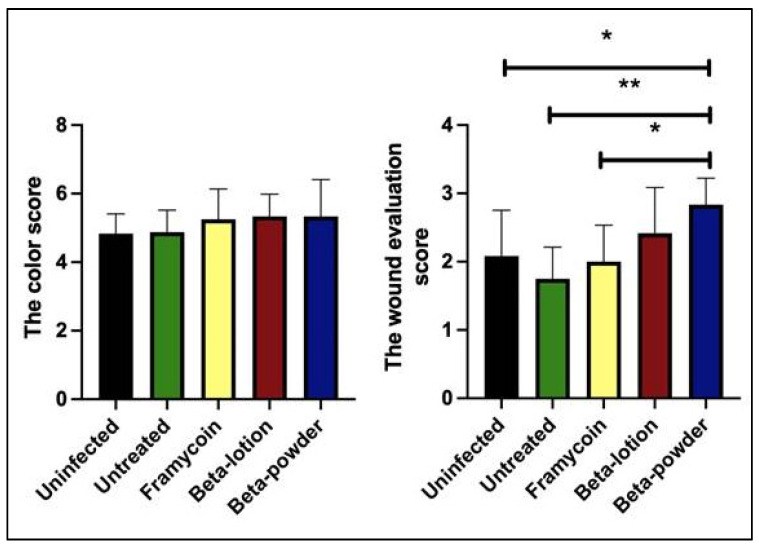
The wound healing evaluation. Individual wound characteristics were assigned numerical scores and the analysis for day 10 is shown. Statistical analysis was performed with the nonparametric Kruskal–Wallis test and the multiple comparison test (* *p* < 0.05 and ** *p* < 0.01).

For more detailed wound assessment, the wound management framework characteristics were recorded and compared for the individual treatment modalities. In general, the signs of ongoing infection were clearly present during the first surgical dressing on day 3. The wounds with MRSA were characterized by hyperemia, redness in the surroundings, and pungent odor. On day 7, however, the wounds with beta-acids showed significant improvement compared to both the negative controls and Framycoin treatment. Wounds with beta-acids showed a decrease in redness, secretion, and odor. On day 10, the wounds treated with beta-acids were filled with fresh granulation tissue. Once again, the most pronounced effects were observed with the powder form of beta-acids. The detailed data for each wound recorded using original scales of the Wound Healing Continuum are available in Table S1 (Supplementary Material). To allow for further statistical analysis the original criteria were assigned numeric scores and the results of the color score and the total score evaluation on day 10 of the experiment are shown in Figure 4.

While the color characteristics alone showed no significant differences between the individual treatments, the application of beta-acids exhibited a clear positive effect on the overall healing score. Beta-acids in powder form show marked and significant differences when compared to the untreated and Framycoin-treated wounds. Interestingly, the overall healing was improved even in comparison to the uninfected wounds, indicating a potential effect on the wound healing per se, not only in connection to its anti-microbial effects.

**Figure 5:**
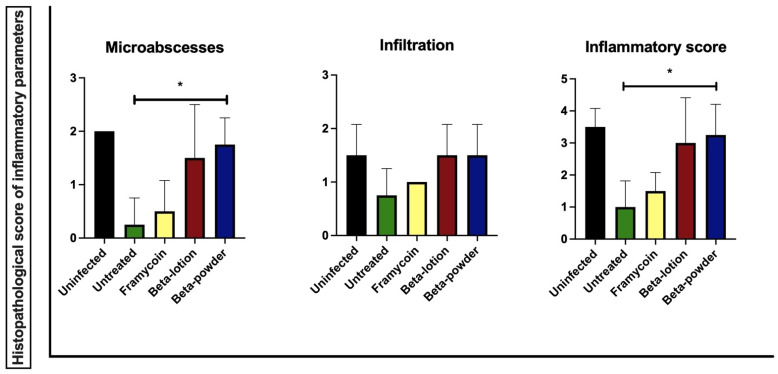
The histopathological score of the inflammatory response. The statistical analysis was performed with the parametric one-way ANOVA test with post hoc Dunn’s comparison test (* *p* < 0.05).

Finally, the effects of beta-acids on the MRSA-infected wounds were assessed on the microscopic level. Tissue samples from the wounds were processed as indicated in the Materials and Methods section, and the following categories of parameters were scored: epithelial tissue recovery, connective tissue recovery, and inflammatory parameters. The results of the effect of the treatments on the individual inflammatory parameters are shown in Figure 5.

**Figure 6:**
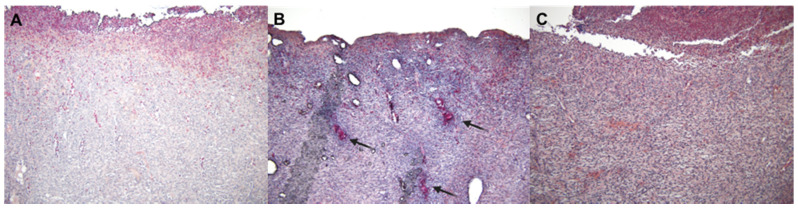
Histopathological changes in the wounds infected with MRSA. Naphthol AS-D chloroacetate-stained samples of porcine skin taken from the center of wounds, 100-fold the original magnification. (**A**) Non-infected samples with mild granulocyte infiltration underneath a fibrin clot. (**B**) Infected wound receiving no treatment with three microabscesses (indicated by arrows) and generally moderate granulocyte infiltration in the surrounding connective tissue. (**C**) Infected wound treated with beta-acid powder, showing mild granulocyte infiltration underneath a fibrin clot.

The data show that beta-acids in both application forms exhibit distinct positive effects on microabscess formation and total inflammation score compared to the untreated wounds. These effects were statistically significant when beta-acids were applied in the powder form (*p* < 0.05). Representative microphotographs of the inflammatory changes in wounds after the infection and treatment with beta-acids are shown in Figure 6.

No effects of the infection or individual treatments were observed in the epithelial and connective tissue recovery parameters (Supplementary Figures S1–S5).

## 3. Discussion

Bacterial skin and soft tissue infections represented by impetigo, folliculitis, furuncle, carbuncle, erysipelas, cellulitis, fasciitis, and myonecrosis are frequently caused by *Staphylococcus aureus*. Kolar et al. proved that the most prevalent etiological agent of infected decubitus ulcers, leg ulcers, and bacterial infections of surgical wounds was *S. aureus* with an MRSA frequency of 9% [16]. At the same time, MRSA is one of the most common nosocomial pathogens with a worldwide distribution. Because of its higher resistance against regularly used antibiotics, the research of effective drugs with antimicrobial properties are still necessary. Several new antibiotics were developed in the past 20 years, which are active against certain strains of MRSA and also present a potential in the treatment of wounds (reviewed in [17]). Non-healing wounds currently represent one of the most common problems encountered in healthcare systems and hospitals. The healing process is often complicated by wound infection with nosocomial bacterial strains, which may lead to the development of chronic wounds requiring long-term treatment [18]. Given this situation, there is an urgent need for useful therapeutics providing both a healing effect and antimicrobial activity. The use of topical antibacterial treatments has clear advantages in the treatment of infected wounds [19]. However, while the antibiotic resistance of the bacteria is still on the rise, the antibiotic research and development has been stalled due to commercial and regulatory reasons [20,21,22].

Due to all the reasons above, there has been an increasing interest in recent years in compounds derived from plants and herbs for their medicinal properties and anti-microbial activities [17]. Thus, for example, tea tree oil and manuka honey have been shown to exert positive effects on healing of MRSA-infected wounds as adjuvant therapeutics improving the effect of antibiotics [23,24]. Numerous studies have shown that the hop cones represent an abundant source of components with apparent antimicrobial effects against selected strains of bacteria, viruses, fungi, and protozoa. One of the specific properties for the use of hop derivatives as therapeutics is their low cytotoxicity. These features lead to their safety and the absence of side effects. The antibacterial properties of some purified compounds from hops, namely xanthohumol and beta-acids, were determined for many pathogens (including MRSA) in vitro in the past [5,6,13].

The standard approach of testing individual compounds from natural sources rarely produces clinically valuable products, and potent activity against planktonic bacteria in a standard lab media rarely translates to the in vivo efficacy. Numerous studies have used porcine models to evaluate the therapeutic effects of novel compounds, treatments, or devices on wound healing processes [25,26,27,28].

In the presented study, the efficiency of the treatment with hop-isolated beta-acids was tested in the porcine model of external wounds infected with MRSA. We first successfully determined the antimicrobial activity of tested compounds against this pathogen in vitro under the conditions simulating the wound environment. The antimicrobial activity of xanthohumol on plates with 3% BSA content was no longer detectable. The antimicrobial effects of beta-acids were still detectable, but at much higher concentrations than the MICs detected using the standard conditions [13]. The effect was seen at all three concentrations used with no clear dose-dependent effects. This suggests that binding of beta-acids from hops to the proteins affects their antimicrobial properties, and therefore, the concentrations in the in vivo treatments have to be adjusted accordingly.

The in vivo activity of beta-acids was tested using a porcine model of external wounds that were previously infected with MRSA. Two modes of application of the active compounds have been used in this study—the lotion with Ambiderman base and the powder form—to investigate whether the mode of application influences the effect in the wounds. Ambiderman was selected based on our preliminary results from the in vitro tests, where it showed good release parameters for the hop compounds among the nine different bases tested (data not shown). The individual modes required differential approaches in the application and choice of dressings to ascertain that the compounds were uniformly spread in the wound and did not leak out between the individual dressings. Thus, beta-acids in lotion were applied in a standardized volume to uniformly fill each wound and covered with standard Curad dressings. The powder form was spread on each wound uniformly by a sterile loop and then covered with Polymem pads. These are non-adherent and non-absorbent polyurethane pads frequently used as dressings of skin wounds in surgery in combination with antibacterial substances [29,30]. As such, these pads prevent the leakage of the powdered substance out of the wound and its absorption into the dressing. Regardless of the application form, the amount of the active substance per wound was kept uniform to allow for direct comparisons. Both application forms of beta-acids exhibited strong antimicrobial properties against the MRSA infection in vivo. This was manifested by both the reduction of MRSA in the wounds and a decrease in inflammation when compared to the untreated controls. However, the powder form of beta-acids has shown both stronger antimicrobial effects and healing potential, which were statistically significant. This may be in part due to the mode of application, where the powder is applied to the wound and covered by a non-adherent dressing.

This study illustrates the antimicrobial and therapeutic potential of beta-bitter acids in wound healing with MRSA contamination. The obtained results show a potential of these compounds to be developed into an antimicrobial treatment scheme or to be used in combination with standardly used drugs. In fact, having all the following properties, beta-acids could represent an ideal topical antimicrobial for the use in chronic wounds: targeted antimicrobial spectrum, rapid and persistent activity allowing for infrequent dosing, low local absorption, activity in the presence of exudate, and low cost [31].

## 4. Materials and Methods

### 4.1. Hop Compounds

Pure beta-acids of hops were isolated from CO_2_ extract (variety Magnum; Hopsteiner, Germany) by two purification steps according to schedule elaborated by the Hop Research Institute [32]. The first step involved partitioning of the extract solution in toluene in an alkaline medium of disodium carbonate (c = 0.2 mol∙L^−1^) and sodium hydroxide (c = 1 mol∙L^−1^). In the second step, crude extract of beta-acids containing up to 3% *w/w* of residual alpha-acids was recrystallized from the mixed solvent acetonitrile/water. The final preparation of the beta-acids was entirely free of alpha-acid residues. Residual solvent was removed by incubation at 40 °C and beta-acids of the minimal purity of 98% were then reconditioned in a refrigerator for 24 h and kept at −18 °C until further use. The lotions containing 3%, 5%, and 10% (*w/w*) of xanthohumol or beta-acids were prepared by mixing the purified compounds with hydrophilic base Ambiderman (containing 8 g of paraffinum liquidum, 12 g of paraffinum solidum, 2 g of alcohol stearilicus, 5 g of propylenglycolum, 2 g of slovasol 2430, 0.5 g of carbomera, 0.6 g of trolaminum, 0.2 g of methylparabenum, 0.05 g of propylparabenum, and 69.65 g of aqua purificata in 100 g). This formulation was previously shown to be effective in vitro [13].

### 4.2. Bacterial Strain and Culture Conditions

The methicillin-resistant *S. aureus* used in this study was from the collection of isolates of the Department of Microbiology of University Hospital in Olomouc (Czech Republic). The strain was cultured on blood agar, Mannitol Salt Phenol Red agar and Oxacillin Resistance Screening Agar Base (all from LabMediaServis, Jaromer, Czech Republic). Bacterial stocks for cryopreservation were prepared on porous beads (ITEST, Hradec Kralove, Czech Republic). For each experiment, fresh bacterial culture was prepared by inoculation of the porous bead with MRSA onto the blood agar plate. The subsequent culture was performed under aerobic conditions at 37 °C for 24 h.

Quantitation of bacterial growth in the wounds was performed by a semiquantitative culture analysis [33,34,35,36]. The swab samples from the wounds were inoculated onto agar plates. Then, the inocula were spread further using subsequent streaks of the loop progressing from quadrant 1 of the plate (the swab inoculum) towards quadrants 2, 3, and 4 in a step-wise fashion. The results were then reported as 1+, 2+, 3+, and 4+, indicating the number of the quadrant where the presence of bacterial growth was still detected, or as 0 for no apparent growth. The number of the quadrant with a detected growth directly correlates to the quantity of the bacteria in the inoculum.

### 4.3. In Vitro Antimicrobial Testing of Hops Compounds

The evaluation of antimicrobial activities of tested compounds from hops was performed using a modified protocol of agar diffusion test. Briefly, the bacterial inoculum in a physiologic solution with a turbidity of 0.5 degrees of the McFarland scale was prepared from a pure bacterial culture incubated for 24 h. Subsequently, the Mueller-Hinton agar containing culture dishes supplemented with 3% bovine albumin (BSA) were inoculated by streaking with the swab containing the inoculum. After 3–5 min of drying, the wells for the tested compounds were prepared using a sterile puncher. The distance of the center of each well from the edge of the petri dish was 24 mm and no closer than 10 to 15 mm. Afterward, each individual tested substance was applied into the wells at concentrations 3%, 5%, and 10% in a volume of 200 µL in three replicates. The agar plates were then incubated at 37 °C for 24 h and the diameter of zones of inhibition was recorded using a standardized ruler.

### 4.4. Animals and Housing

Six experimental adult female pigs (*Sus scrofa f. domestica*, hybrid of Czech White and Landrace breeds; weight mean 50 ± 0.3 kg) were enrolled into the study. The pigs were housed in an accredited vivarium (temperature 21 ± 1 °C, naturally light/dark cycle). All animals were fed with standard assorted A1 food (VKS Pohledecti Dvoraci, Havlickuv Brod, Czech Republic) in equal amounts twice a day and had free access to drinking water. The acclimatization period was 7 days before the experiment. The project was approved by the Institutional Review Board of the Animal Care Committee of the University of Defence (record number MO 54549/2017–684800 and MO 103191/2018–684800), Faculty of Military Health Sciences, Hradec Kralove, Czech Republic. Animals were treated in accordance with the European Convention for the Protection of Vertebrate Animals and in accordance with the ARRIVE Guidelines [37]. All workers who manipulated animals are holders of a Certificate of Professional Competence to Design Experiments and Experimental Trials under the Animal Welfare.

### 4.5. Experimental Model

**Figure 7:**
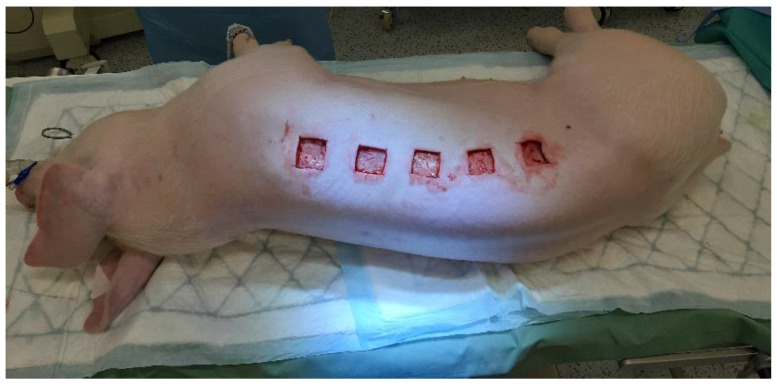
The creation of skin wounds.

The animals were anesthetized 30 min before start of the procedure with 30 mg/kg of ketamine (Narkamon, Bioveta, Ivanovice na Hane, Czech Republic), 40 mg∙kg^−1^ azaperone (Stressnil, Janssen Pharmaceutica, Beerse, Belgium), and 0.05 mg∙kg^-1^ of atropine (Atropin Biotika, Biotika Bohemia, Prague, Czech Republic), using intra-muscular injection. An intravenous application of propofol (Fresenius Kabi AG, Bad Homburg, Germany) was used for the subsequent maintenance of general anesthesia. The dorsal and lateral thorax of pigs were clipped, washed with an antimicrobial-free soap, and shaved with a razor. Each animal was intubated and prepared for surgery using isopropyl alcohol to disinfect the skin surface. The site of the wound creation was designated with a marking pen on the skin over the dorsal muscle of the pig. Ten deep surgical wounds were aseptically created by the scalpel using a sterile stainless steel template with the internal dimensions of 3.5 × 3.5 cm (Figure 7).

The wounds penetrated the level of the muscular fascia (approximately 0.5 cm deep). The wounds were separated by 30 mm of unwounded skin in between. Eight wounds were then inoculated with 0.1 mL of MRSA suspension at uniform concentrations of 9 × 10^5^ CFU per mL and allowed to sit undisturbed for 2 min. Two non-infected wounds treated with 0.1 mL sterile saline solution were used in the study as a negative control. All wounds were covered with sterile compress. The wounds subsequently treated with the powder form of beta-acids were covered by non-adherent Curad dressings (Medline, Northfield, Minnesota), two layers of Omnifix Elastic (Hartmann Rico, Veverska Bityska, Czech Republic), and the entire animal loosely wrapped with elastic wrap. After each procedure, the general appearance of the animals was followed, including the willingness to eat and drink, signs of lameness or pain, and signs of systematic disease.

### 4.6. Wound Treatments and Macroscopic Assessment

**Figure 8:**
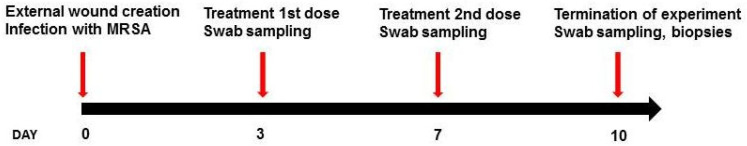
The timeline of the experiment.

In all of the experiments, two wounds on each animal were established as negative controls (infection-free). The rest of the wounds were infected by MRSA suspension at uniform concentrations (see experimental model). The timeline of the experiment is provided in Figure 8.

On days 3 and 7 after the wound creation, the antibacterial compounds were applied during surgical dressings. Framycoin (active substances: zinc bacitracin 2500 IU, neomycin sulfas 52 mg in 10 g of ointment) in the amount of 1.5 g∙wound^−1^ was used in two wounds on each animal as a positive control. The wounds were, thus, subsequently analyzed in the following categories—uninfected (*n* = 12), infected with no treatment (*n* = 8), Framycoin application (*n* = 8), application of beta-acids in powder form (*n* = 12), and in Ambiderman base (*n* = 12). The amount of beta-acids applied was kept uniform at 300 mg∙wound^−1^. Thus, the following procedures were used in the respective wounds: 300 mg∙wound^−1^ of beta-acid powder were applied and uniformly spread on the wound surface by a sterile loop, while 3 mL of 10% beta-acid in Ambiderman base were applied to fill the wound. All dressings were performed in general anesthesia as follows. The animals were anesthetized 30 min before the procedure with 30 mg∙kg^−1^ of ketamine (Narkamon, Bioveta) and 0.05 mg∙kg^−1^ of atropine (AtropinBiotika, Biotika Bohemia), using intra-muscular injection. An intravenous application of propofol (Fresenius Kabi AG) was used for subsequent maintenance of general anesthesia. Before the first application of the hop substances on day 3, bacterial swab samples were taken from all wounds to evaluate the magnitude of MRSA infection. The wounds were macroscopically evaluated and pictures were taken.

After that, the active substances were applied to the wounds. The beta-acids in lotion filled the entire wound. The wounds were then covered with sterile compresses 5 × 5 cm and with two layers of Omnifix Elastic. The beta-acids in the powder form were dispersed over the whole wound area, after which the wound was filled with Polymem pads and covered with sterile compresses 5 × 5 cm and two layers of Omnifix Elastic. Wounds with Framycoin lotion were covered with compresses 5 × 5 cm and covered with two layers of Omnifix Elastic.

On day 7, the dressings and substance applications were performed in a similar manner, including the wound examinations. In the wounds with the lotion small irrigation with physiological saline solution was necessary to remove the dressing without wound irritation. On day 10 after the wound creation, the experiment was terminated: the last swabs were taken and, following the euthanasia, cross-sections of the wounds were sampled for histopathology.

During each surgical dressing the wound healing process was scored using The Wound Healing Continuum (color-based continuum) [15]. Briefly, this system scores selected criteria of wound appearance using colors (Black, Black-Yellow, Yellow, Yellow-Red, Red, Red-Pink, Pink) and the presence or absence of wound secretions, odor, and redness. A black wound base indicates a necrotic tissue with no healing potential. A yellow wound presents adherent fibrous material, which together with wound exudate is an ideal medium for bacterial growth and ongoing infection. The Red and Red-Pink tissue presents new vessel growth, granulation tissue matrix, and indicates wound healing.

### 4.7. Histopathology Examination

Collected samples were fixed in 10% neutral buffered formalin (Bamed, Ceske Budejovice, Czech Republic). Subsequently, they were histologically processed and stained with hematoxylin and eosin (both from Merck, Kenilworth, NJ, USA), according to Pejchal et al. [38]. Additionally, hydrated samples were stained with naphthol AS-D chloroacetate (specific esterase) Kit (Sigma-Aldrich, St. Louis, MO, USA) according to the manufacturer’s instructions and with Masson’s trichrome staining according to the procedure published previously [39].

Histopathological parameters were evaluated using an Olympus BX51 microscope (Olympus, Tokyo, Japan) by a histopathologist who was blinded to the treatment. The total histopathological score was defined as the sum of the nine parameters described in Table 2.

These parameters included healing of the epithelial layer (extent of newly formed epithelial layer and its predominant differentiation), healing of connective tissue (amount of granulation tissue, collagen fiber orientation, collagen fiber pattern, amount of early collagen, and amount of mature collagen), and the inflammatory response (inflammatory infiltrate, presence of microabscesses). Parameters 1, 2, and 3 were evaluated from hematoxylin-eosin-stained samples. Masson’s trichrome-stained samples were used to measure parameters 4–7. Finally, naphthol AS-D chloroacetate-stained samples helped to score parameters 8 and 9. All these indicators were evaluated only in the area covering one microscopic field at 200-fold magnification beneath the fibrin or epithelial layer from the right to the left edge of the wound (at the surface).

### 4.8. Data Analysis

Data of wound monitoring were analyzed using Excel (Microsoft^®^ 2010) software or imported into GraphPad Prism 9 (version 9.1.0, GraphPad Software Inc., San Diego, CA, USA) for further analysis. The normality was tested using the Shapiro–Wilk test. Normally distributed data were analyzed using a one-way ANOVA with post-hoc test. Non-normally distributed data were analyzed by a Kruskal–Wallis test with multiple comparison test. The differences were considered significant when *p* ≤ 0.05.

## 5. Conclusions

Taken together, the data from our porcine model of the in vivo infection of MRSA-infected wounds show clear effects of beta-acids from hops on both the reduction of bacterial load in the wounds and improvement of macroscopic as well as microscopic parameters of wound healing. The powder application form of beta-acids shows superior results to those obtained with the active compound in the Ambiderman base. The presented study determined in the animal model that the beta-bitter from hop acids significantly improve both the wound healing process and antibacterial activity. Thus, these compounds could have potential for human application.

## 6. Patents

Bostik P, Sleha R, Houska M, Mikyska A, Krofta K inventors. Lecebna latka z chmele pro tezce se hojici rany jako nahrada antibiotik a zpusob jeji aplikace (Medicinal substance from hops for difficult-to-heal wounds as a substitute for antibiotics and the method of its application). Czech patent application number PV 2021–195. Industrial Property Office of the Czech Republic. Apr 19, 2021. Czech

Bostik P, Sleha R, Houska M, Mikyska A, Krofta K inventors. Lecebna latka z chmele pro tezce se hojici rany jako nahrada antibiotik a zpusob jeji aplikace (Medicinal substance from hops for difficult-to-heal wounds as a substitute for antibiotics and the method of its application). Czech utility model application number PUV 2021–38741. Industrial Property Office of the Czech Republic. 19 April 2021. Czech

## Figures and Tables

**Table 1:** The antimicrobial activity of hops compounds against the MRSA strain obtained by the agar diffusion method.

Tested Compound	Diameter of Inhibition Zone (mm) ^1^		
	3%	5%	10%
Beta-acids	11.2 ± 1.1	11.6 ± 1.3	12.8 ± 0.8
Xanthohumol	0	0	0
Ambiderman	0	0	0

^1^ Data are presented as mean ± standard deviation of inhibition zone diameters (*n* = 5).

**Table 2:** Histopathological parameters of wound healing.

Score	0	1	2	3
**Healing of Epithelial Layer**				
Extent of the newly formed layer	Does not Reach the Cutting Edge	On the Cutting Edge	≤50% of the Wound ^a^	≥50% of the Wound ^a^
Differentiation	none	spinous	granular	
**Healing of Connective Tissue**				
Amount of Granulation Tissue	profound	moderate	scanty	absent
Collagen Fiber Orientation	vertical	mixed	horizontal	
Collagen Fiber Pattern	reticular	mixed	fascicle	
Amount of Early Collagen	profound	moderate	minimal	absent
Amount of Mature Collagen	absent	minimal	moderate	profound
**Inflammatory Response**				
Inflammatory Infiltrate	Plenty	Moderate	Mild	A few
Presence of MicroAbscesses	≥2	1	none	

^a^ does not include the cutting edge.

## Data Availability

Data are available on request due to ethical restrictions.

## Supplementary Materials

The following are available online at https://www.mdpi.com/article/10.3390/antibiotics10060708/s1, Table S1, The original wound healing evaluation scores; Figure S1, The histopathological score of epithelial tissue parameters; Figure S2, The histopathological score of connective tissue recovery; Figure S3, The total histopathological score; Figure S4, Histopathological changes in the wounds infected with MRSA; Figure S5, Histopathological changes in the wounds infected with MRSA.
