# Supplementary material for: Strong Antimicrobial and Healing Effects of Beta-Acids from Hops in Methicillin-Resistant Staphylococcus aureus-Infected External Wounds In Vivo
Source: Antibiotics (Basel). 2021 Jun 12;10(6):708. doi: 10.3390/antibiotics10060708 (PMC8231114; doi:10.3390/antibiotics10060708)
Supplement: Supplementary file 1 [file antibiotics-10-00708-s001.zip › antibiotics-1241049-supplementary.pdf]

antibiotics

MDPI

Online Supplemental Material to:

Article

Strong Antimicrobial and Healing Effects of Beta-Acids from Hops in Methicillin-Resistant *Staphylococcus aureus*-Infected External Wounds In Vivo

Radek Sleha [1], Vera Radochova [1], Jiri Malis [1,2], Alexander Mikyska [3], Milan Houska [4], Karel Krofta [5], Katerina Bogdanova [6], Sylva Janovska [1], Jaroslav Pejchal [7], Milan Kolar [6], Pavel Cermak [2] and Pavel Bostik [1,8,*]

[1] Department of Epidemiology, Faculty of Military Health Sciences, University of Defence, 50001 Hradec Kralove, Czech Republic; radek.sleha@unob.cz (R.S.); vera.radochova@unob.cz (V.R.); jiri.malis@ftn.cz (J.M.); sylva.janovska@unob.cz (S.J.)
[2] Thomayer Hospital, 110 00 Prague, Czech Republic; pavel.cermak@lf3.cuni.cz
[3] Research Institute of Brewing and Malting, 110 00 Prague, Czech Republic; mikyska@beerresearch.cz
[4] Food Research Institute, 110 00 Prague, Czech Republic; milan.houska@vupp.cz
[5] Hop Research Institute, 438 01 Zatec, Czech Republic; krofta@chizatec.cz
[6] Department of Microbiology, Faculty of Medicine and Dentistry, Palacky University, and University Hospital, 771 47 Olomouc, Czech Republic; katerina.bogdanova@fnol.cz (K.B.); milan kolar@fnol.cz (M.K.);
[7] Department of Toxicology and Military Pharmacy, Faculty of Military Health Sciences, University of Defence, 50001 Hradec Kralove, Czech Republic; jaroslav.pejchal@unob.cz
[8] Institute of Clinical Microbiology, Faculty of Medicine in Hradec Kralove, Charles University and University Hospital, 50005 Hradec Kralove, Czech Republic
* Correspondence: pavel.bostik@unob.cz

The assessment of wound healing parameters.

To evaluate the individual wounds during the course of the experiment from the perspective of healing proces the Wound Healing Continuum was used [15].

The following categories were assessed: Color, Redness, Odor, Wound secretion. After the evaluation, individual values of each category were assigned a numerical score to allow for further statistical evaluation

In the individual categories the values were as follows (the assigned numeric score is in parentheses):

Color: B - Black (0), BY - BlackYellow (1), Y - Yellow (2), YR - YellowRed (3), R - Red (4), RP - RedPink (5), P - Pink (6)

Redness: + presence (1), - absence (0)

Odor: + presence (1), - absence (0)

Wound secretion: + presence (1), - absence (0)

The values for the individual wounds are presented in the Supplementary Table 1.

Table S1. The original wound healing evaluation scores.

Group	Day	Wound 1	Wound 2	Wound 3	Wound 4	Wound 5	Wound 6	Wound 7	Wound 8	Wound 9	Wound 10	Wound 11	Wound 12
	3	RP/-/-/+	RP/-/-/+	RP/-/-/+	RP/-/-/+	RP/-/-/+	RP/-/-/+	RP/-/-/+	RP/-/-/+	RP/-/-/+	RP/-/-/+	RP/-/-/+	RP/-/-/+
**Uninfected**	7	RP/-/-/+	R/-/-/+	RP/-/-/+	R/+/-/+	R/+/+/+	R/+/-/+	YR/-/-/+	R/+/-/-	P/-/-/+	RP/+/-/-	R/+/-/+	R/+/-/+
	10	R/-/-/-	RP/+/-/-	P/-/-/-	RP/-/-/+	RP/-/-/+	R/-/-/+	R/+/-/+	RP/-/+/-	RP/-/-/+	RP/-/-/-	RP/+/-/+	RP/+/-/-
	3	R/+/+/+	R/+/+/+	R/+/+/+	R/+/+/+	R/+/+/+	R/+/+/+	R/+/+/+	R/+/+/+				
**Untreated**	7	RP/+/+/+	RP/+/+/-	RP/+/+/+	R/+/+/+	RP/+/+/+	R/+/+/-	R/+/-/+	RP/+/+/+				
	10	RP/+/-/-	RP/+/-/+	RP/+/-/+	P/-/-/+	RP/-/+/-	R/-/+/-	R/-/-/+	RP/+/-/-				
	3	R/+/+/+	R/+/+/+	R/+/+/+	R/+/+/+	R/+/+/+	R/+/+/+	R/+/+/+	R/+/+/+				
**Framycoin**	7	RP/+/+/+	R/+/+/+	R/+/+/+	YR/+/+/+	RP/+/+/+	R/+/-/-	R/+/+/+	R/+/+/+				
	10	P/+/-/-	P/-/-/+	P/-/-/+	RP/+/-/+	P/-/+/-	RP/+/-/-	R/-/-/-	R/+/-/-				
	3	R/+/+/+	R/+/+/+/	R/+/+/+	R/+/+/+	R/+/+/+	R/+/+/+	R/+/+/+	R/+/+/+	R/+/+/+	R/+/+/+	R/+/+/+	R/+/+/+
**Beta-lotion**	7	RP/+/+/+	RP/+/+/+	RP/+/+/+	RP/+/+/+	R/+/+/+	RP/+/-/+	P/+/+/+	R/+/+/+	R/+/+/+	R/+/-/-	P/-/-/-	R/+/+/+
	10	RP/+/-/+	RP/+/-/-	RP/+/-/-	RP/-/-/-	P/-/-/-	RP/-/-/-	P/-/-/-	RP/-/-/-	P/-/+/-	R/-/-/-	P/-/-/+	P/+/-/-
	3	R/+/+/+	R/+/+/+	R/+/+/+	R/+/+/+	R/+/+/+	R/+/+/+	R/+/+/+	R/+/+/+	R/+/+/+	R/+/+/+	R/+/+/+	R/+/+/+
**Beta-powder**	7	R/+/+/+	R/+/+/+	R/+/+/+	RP/+/-/+	R/+/+/+	RP/+/-/+	YR/+/+/+	R/+/+/+	YR/+/+/+	RP/+/-/-	P/-/-/-	YR/+/+/+
	10	YR/-/-/-	R/-/-/-	R/-/-/-	RP/+/-/-	P/-/-/-	P/-/-/-	P/-/-/+	P/-/-/-	P/-/-/-	P/-/-/-	P/-/-/-	P/-/-/-

The individual values for each category are showed for each wound in 5 treatment groups on days 3, 7 and 10 in the following order: Color/Redness/Odor/Wound secretion.

Histopathological analysis

Tissue samples from the wounds were processed as indicated in the Materials and Methods and the following categories of parameters were histopathologically scored using a semiquantitative scale: epithelial tissue recovery, connective tissue recovery and inflammatory parameters. Significant differences between individual treatments were observed only for inflammatory parameters and are presented in the main text (Figure 7). Parameters of the epithelial tissue and connective tissue recovery, which either showed minimal effects of the infection or non-significant effects of the individual treatments are shown in Figures S1 and S2 below. Figure S3 presents the total histopathological scores for the wounds in each treatment groups, which were calculated as means of the individual scores in all three categories.

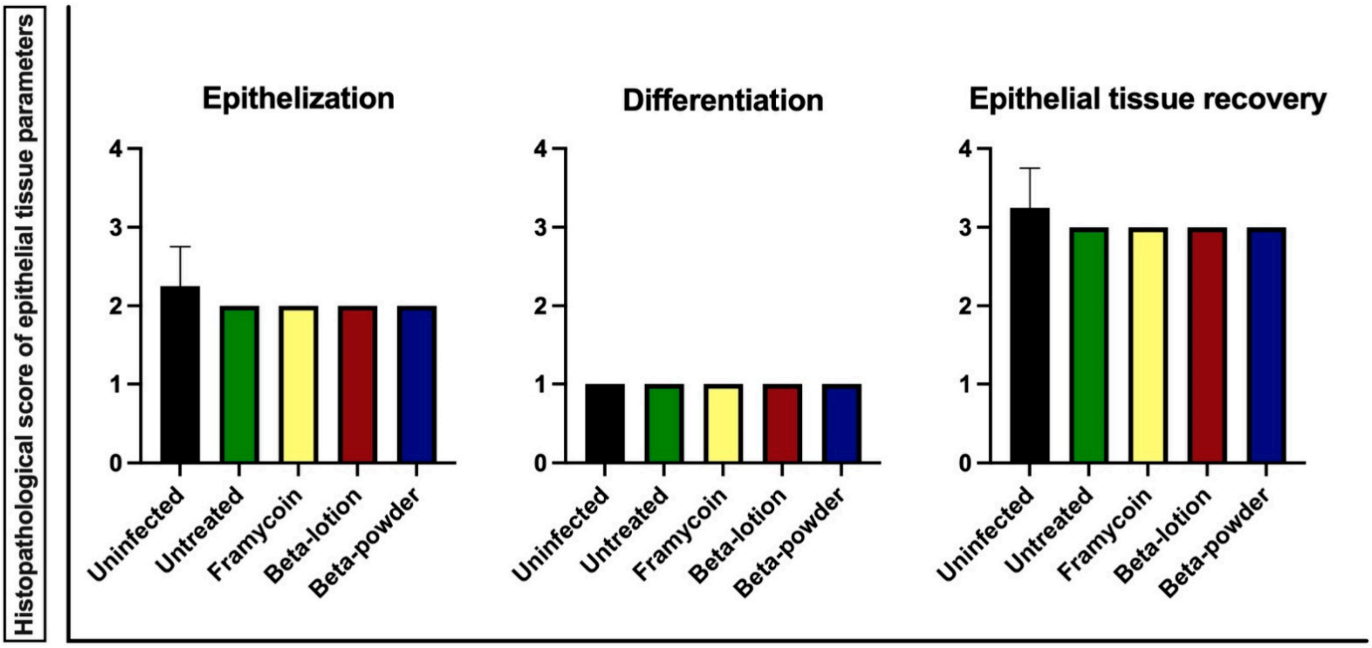

Figure S1. The histopathological score of epithelial tissue parameters. The statistical analysis was performed by the parametric one-way ANOVA test with post hoc Dunn´s comparison test (*p < 0.05).

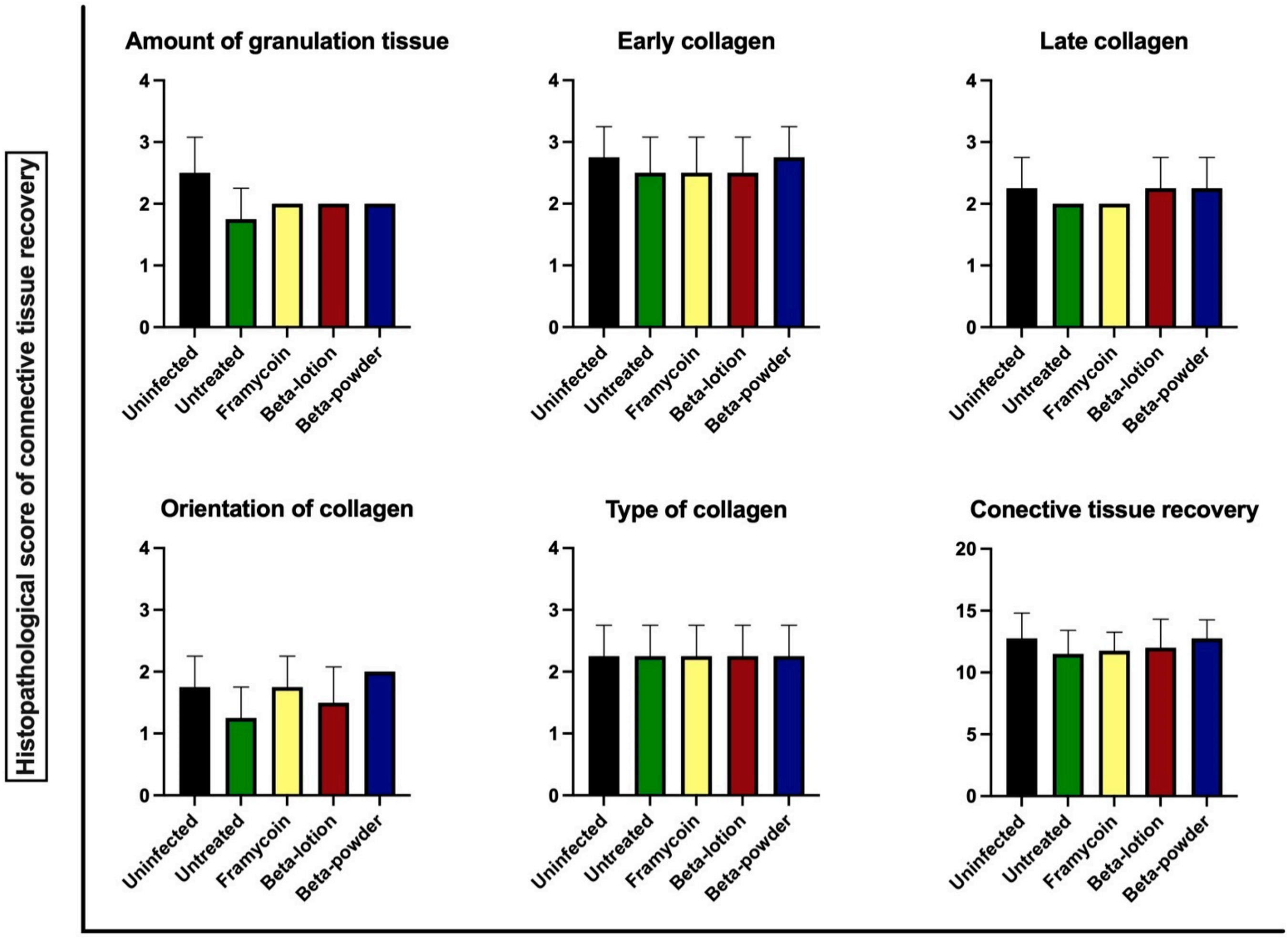

Figure S2. The histopathological score of connective tissue recovery. The statistical analysis was performed by the parametric one-way ANOVA test with post hoc Dunn´s comparison test (*$p < 0.05$).

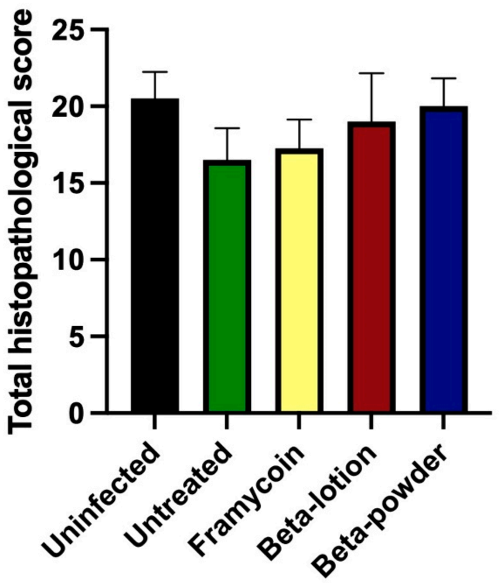

Figure S3. The total histopathological score. The statistical analysis was performed by the parametric one-way ANOVA test with post hoc Dunn´s comparison test (*$p < 0.05$).

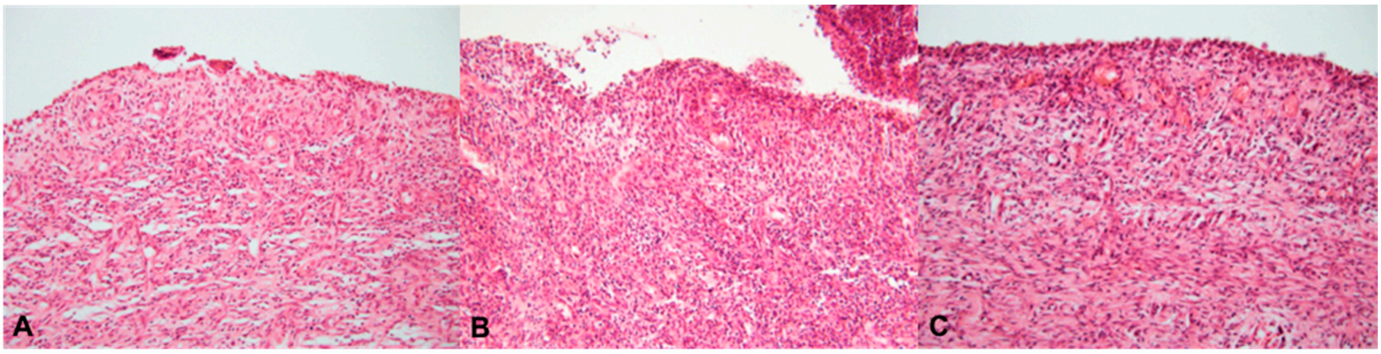

Figure S4. Histopathological changes in the wounds infected with MRSA. Samples obtained from centers of wounds are stained with hematoxylin-eosin and shown at 200fold original magnification. **(A)** the non-infected wound, **(B)** the infected wound with no treatment, and **(C)** the infected wound treated with the beta-acids powder show a moderate amount of granulation tissue.

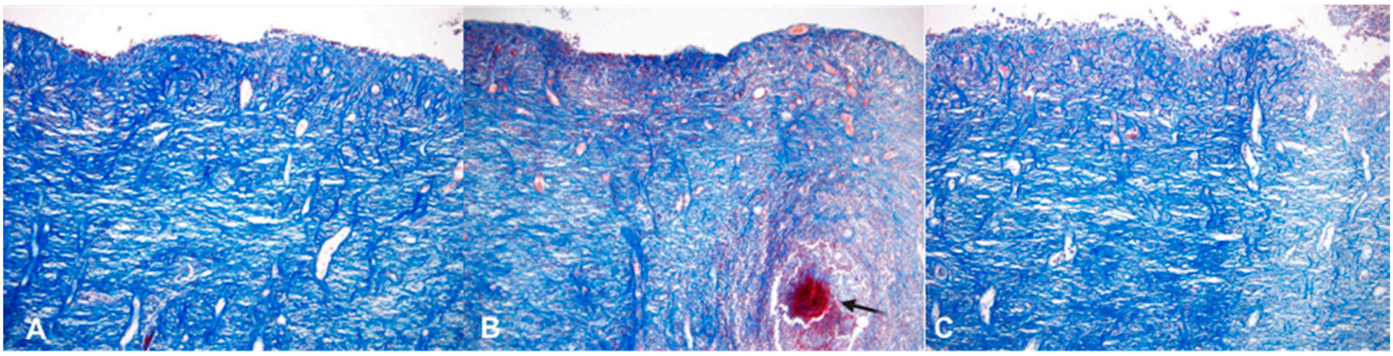

Figure S5. Histopathological changes in the wounds infected with MRSA. Masson`s trichrome-stained samples taken from the center of wounds at 100fold original magnification. **(A)** the non-infected and **(C)** infected wound treated with beta acids powder show a minimal to moderate amount of early (light blue) and mild to moderate amount of mature collagen (dark blue) with mixed collagen fiber orientation and pattern. In **(B)** the infected wound with no treatment, the content of early collagen is slightly increased (moderate amount, i.e. a lower score), while other parameters are not changed compared to both **(A)** and **(C).** The arrow indicates a microabscess.
